# Severe Disseminated Intravascular Coagulopathy Associated With Biventricular Massive Mural Thrombi in Newly Diagnosed Non-ischemic Cardiomyopathy

**DOI:** 10.7759/cureus.39870

**Published:** 2023-06-02

**Authors:** Muhammad Ghallab, Mahmoud Khairy, Allison Foster, Avish Parikh, Giovina Collura

**Affiliations:** 1 Internal Medicine, Icahn School of Medicine at Mount Sinai, New York City (NYC) Health and Hospitals, Queens, New York, USA; 2 Cardiovascular Medicine, Cairo University, Cairo, EGY; 3 Internal Medicine, Queens Hospital Center, New York, USA; 4 Cardiology, Icahn School of Medicine at Mount Sinai, New York City (NYC) Health and Hospitals, Queens, New York, USA

**Keywords:** blood hypercoagulability, hypercoagulability, dilated cardiomyopathy (dcm), mural thrombus, disseminated intravascular coagulation (dic)

## Abstract

Hemostatic system abnormalities have been previously associated with congestive heart failure (CHF). Here, we report a rare case of disseminated intravascular coagulopathy (DIC) in the setting of non-ischemic cardiomyopathy with right atrial and biventricular thrombus. We present a 55-year-old female with a past medical history of bronchial asthma who presented with a six-day history of bilateral leg swelling and dry cough. Her physical examination on admission was significant for signs of biventricular heart failure. Initial workup was significant for elevated pro-brain natriuretic peptide (ProBNP), elevated transaminases, marked thrombocytopenia (19,000/mcL), and coagulopathy with international normalized ratio (INR) of 2.5 and D-dimer of 15,585 ng/mL. Transthoracic echocardiogram (TTE) showed a large mobile right atrial thrombus protruding into the right ventricle and a more adherent left ventricular (LV) thrombus with severely reduced biventricular contractility. Pan CT was done and was significant for multifocal multilobar pulmonary emboli. A lower limb venous duplex was done and revealed extensive bilateral lower limb deep venous thrombosis (DVT). This rare case demonstrates an unusual association between DIC with non-ischemic cardiomyopathy, biventricular thrombus, extensive deep vein thrombosis, and pulmonary embolism (PE). In comparison, there are multiple prior reports for DIC with CHF and LV thrombus. However, our case differs from prior reports in terms of the presence of right atrial and biventricular thrombus. The patient received antibiotics, diuretics, and cryoprecipitate in the setting of persistent low fibrinogen levels. The patient underwent Interventional radiology-guided thrombectomy for extensive pulmonary emboli followed by inferior vena cava (IVC) filter insertion, resulting in the resolution of the right atrial thrombus and extensive decrease of the pulmonary emboli burden. The patient was then given apixaban after normalization of the platelet count and fibrinogen level. Hypercoagulability workup was inconclusive. The patient was then discharged after improvement of symptoms. Early recognition of DIC and cardiac thrombi in patients with new-onset heart failure is crucial for the implementation of the correct management by thrombectomy, optimizing heart failure medications, and anticoagulation to achieve better outcomes.

## Introduction

Hemostatic system abnormalities have been previously associated with congestive heart failure (CHF) [1]. However, disseminated intravascular coagulopathy (DIC) is rarely documented in such patients. DIC is an extensive hypercoagulable state leading to diffuse microvascular and macrovascular clotting, compromising blood flow, and resulting in multiorgan failure. DIC is an uncommon but severe complication of an underlying disease and is most associated with sepsis, trauma, and malignancies. While DIC has been previously reported in patients with acute myocardial infarction [2], there is a lack of literature regarding the association between DIC and non-ischemic cardiomyopathy.

Moreover, a left ventricular (LV) thrombus can complicate both ischemic and non-ischemic cardiomyopathy. However, biventricular thrombi were described in very few cases in the literature [3]. Here, we report a rare case of DIC in non-ischemic cardiomyopathy with right atrial and biventricular thrombi and multiple deep vein thrombosis (DVT) and pulmonary emboli (PE).

## Case presentation

**Figure 1:**
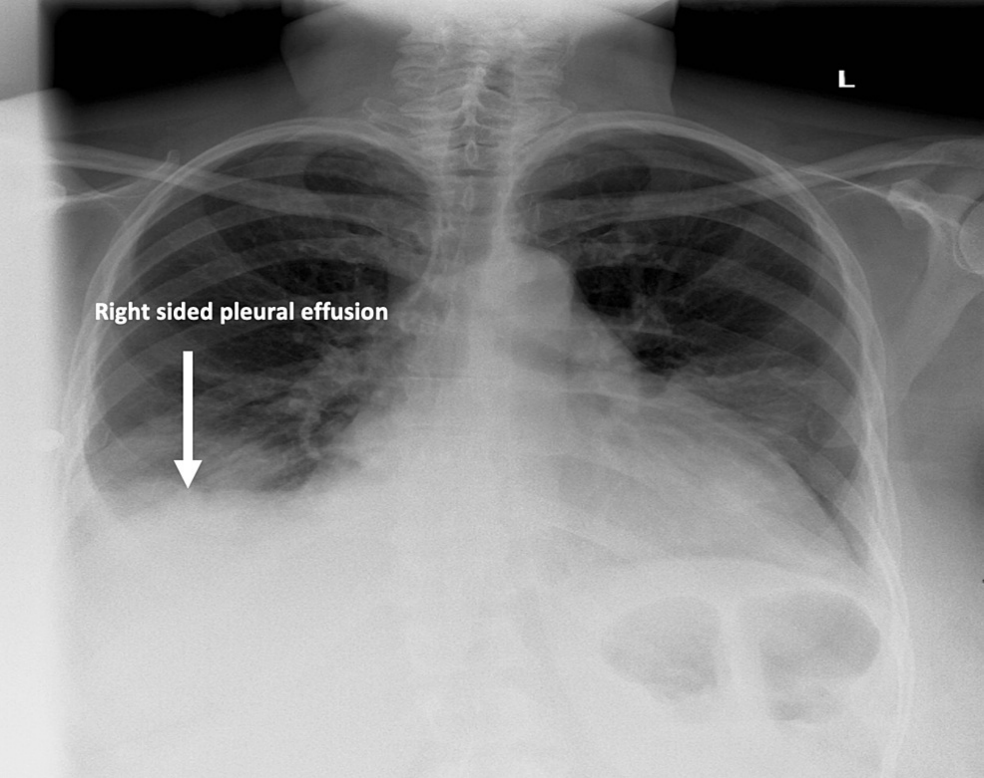
Chest X-ray showing bilateral pleural effusions more pronounced on the right (arrow).

A 55-year-old female with a past medical history of bronchial asthma on an albuterol inhaler as needed presented with a six-day history of bilateral leg swelling and dry cough. Her physical examination on admission was significant for elevated jugular venous pressure, bilateral basal crackles in the lungs, and significant +3 lower limb pitting edema. Initial workup revealed high pro-brain natriuretic peptide (ProBNP) of 17,641 pg/mL (normal: <150 pg/mL), elevated alanine transaminase of 74 U/L (normal: 0-33 U/L), elevated aspartate transaminase of 242 U/L (normal: 5-32 U/L), marked thrombocytopenia of 19,000 × 10^3^/mcL (normal: 150-450 × 10^3^/mcL), elevated C-reactive protein of 73 mg/L (normal: <5 mg/L), coagulopathy with international normalized ratio (INR) of 2.5, prothrombin time of 28 seconds (normal: 11-13 seconds), D-dimer of 15,585 ng/mL (normal: <280 ng/mL), and fibrinogen level of 47 mg/dL (normal: 200-393 mg/dL). Chest X-ray showed bilateral pleural effusions more pronounced on the right (Figure 1). Based on the International Society on Thrombosis and Haemostasis scoring system for DIC, the patient had a score of 8, suggesting a very high probability of overt DIC.

**Figure 2:**
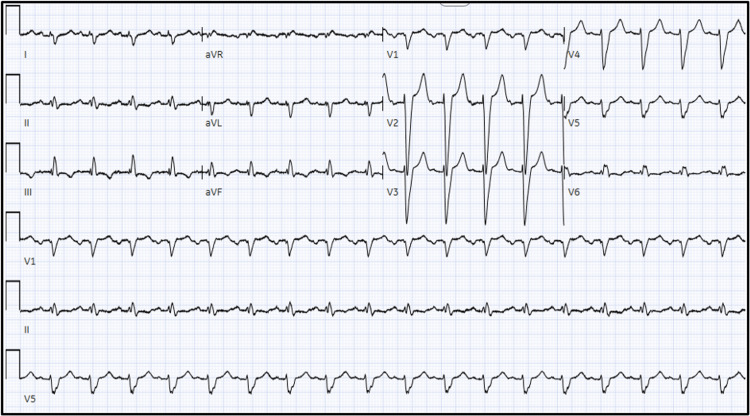
Twelve-lead EKG showing sinus tachycardia, right axis deviation, T-wave inversions in the inferior leads, and intraventricular conduction delay. EKG: electrocardiogram

Electrocardiogram (EKG) showed sinus tachycardia, right axis deviation, T-wave inversions in the inferior leads, and intraventricular conduction delay (Figure 2).

**Figure 3:**
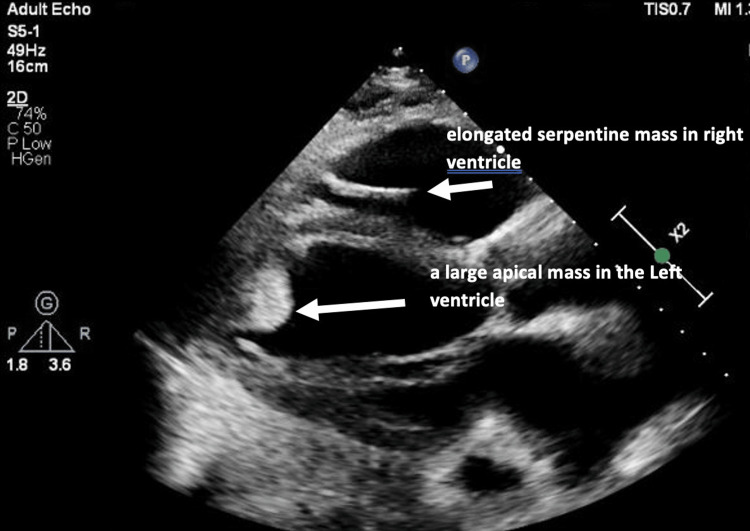
Transthoracic echocardiogram parasternal long-axis view showing a large apical mass in the LV measuring 3.5 × 3 cm (arrow) and elongated serpentine mass in the RV (arrow). LV: left ventricle, RV: right ventricle

Transthoracic echocardiography (TTE) was done and revealed increased LV wall thickness, severely reduced LV (15%), right ventricular (RV) ejection fraction, grade II LV diastolic dysfunction, dilated RV cavity size with reduced RV systolic function, severely dilated left and right atria (RA), and severe tricuspid regurgitation with pulmonary artery systolic pressure (PASP) of 40 mmHg. It also revealed a large thrombus in the LV measuring 3.5 × 3 cm, a large mobile mass in the right atrium intermittently prolapsing through the tricuspid valve (possible thrombus in transit), and a small pericardial effusion (Figure 3 and Video 1, 2).

**Video 1:** Transthoracic echocardiogram parasternal long-axis view showing a large mobile RA thrombus protruding into the RV and a more adherent LV thrombus, with severely reduced biventricular contractility. LV: left ventricle, RV: right ventricle, RA: right atrium

**Video 2:** Transthoracic echocardiogram apical four-chamber view showing large mobile RA thrombus protruding into the RV and an adherent LV thrombus. LV: left ventricle, RV: right ventricle, RA: right atrium

**Figure 4:**
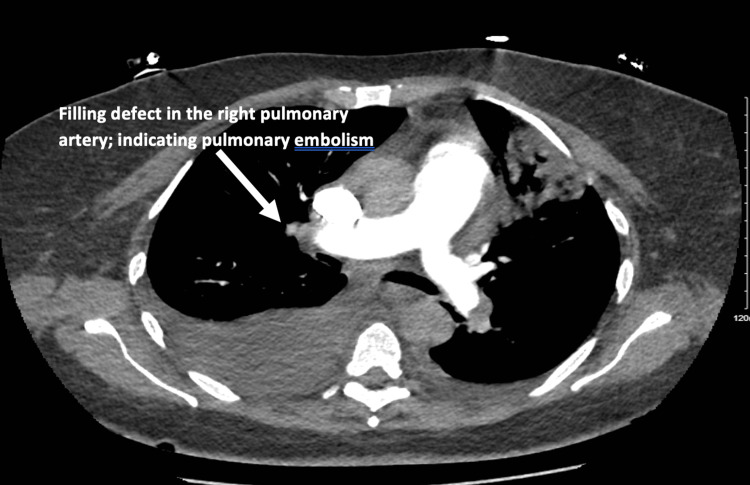
CT pulmonary angiography showing multifocal pulmonary emboli involving the segmental and subsegmental pulmonary arterial vasculature and bilateral pleural effusion (arrow). CT: computed tomography

The patient was started on intravenous (IV) heparin infusion and IV empiric antibiotics for possible sepsis. She was continued on intravenous (IV) furosemide (40 mg twice daily) for volume overload and received additional 10 units of cryoprecipitate in the setting of persistent low fibrinogen level (<100 mg/dL). Pan computed tomography (CT) was done to localize infectious processes or malignancies. CT of the head, abdomen, and pelvis results were negative for infections or malignancies. CT pulmonary angiography revealed multifocal multilobar PE (Figure 4).

**Figure 5:**
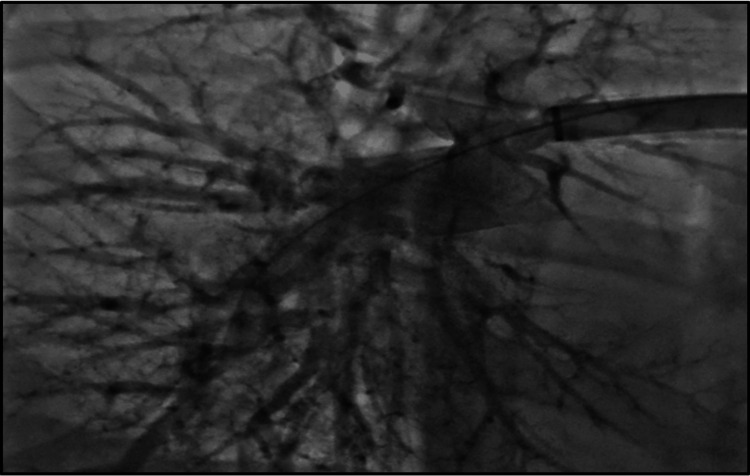
IR-guided thrombectomy of the right pulmonary artery. IR: interventional radiology

**Figure 6:**
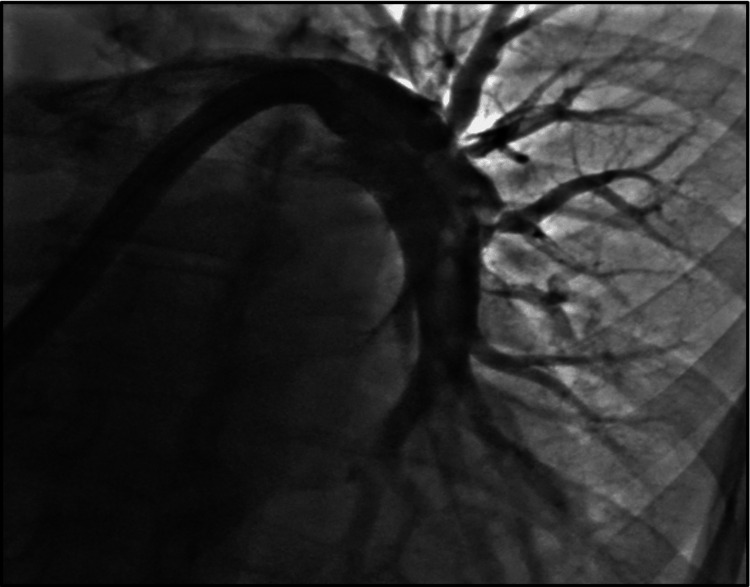
IR-guided thrombectomy of the left pulmonary artery. IR: interventional radiology

**Figure 7:**
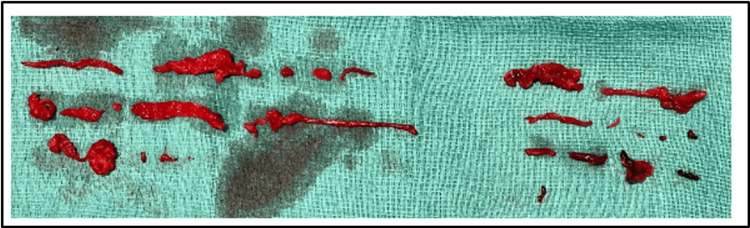
Extensive thrombus burden post-pulmonary artery and RA thrombectomy. RA: right atrium

The patient underwent interventional radiology (IR)-guided thrombectomy with the extraction of thrombi from the RA, left pulmonary artery (PA), and right PA, followed by inferior vena cava (IVC) filter insertion (Figures 5-7). There was no significant improvement in the PA hemodynamics and cardiac index post-thrombectomy, which could suggest underlying chronic cardiac dysfunction and/or chronic pulmonary embolism.

On day 4, the patient remained afebrile with no cough. Infectious disease service follow-up recommended keeping antibiotics coverage because of unclear DIC etiology, with low infectious process suspicion. On day 5, the patient remained hemodynamically stable and afebrile and was then transferred to the step-down unit for further management. Laboratory workup showed continuous improvement. White blood count (WBC) was 9.8 × 10^3^/mcL, D-dimer was 14,183 ng/mL, platelet count was 180 × 10^3^/mcL, fibrinogen level was 168 ng/mL, and prothrombin time (PT) was 15.9 seconds. Blood culture results were negative.

**Figure 8:**
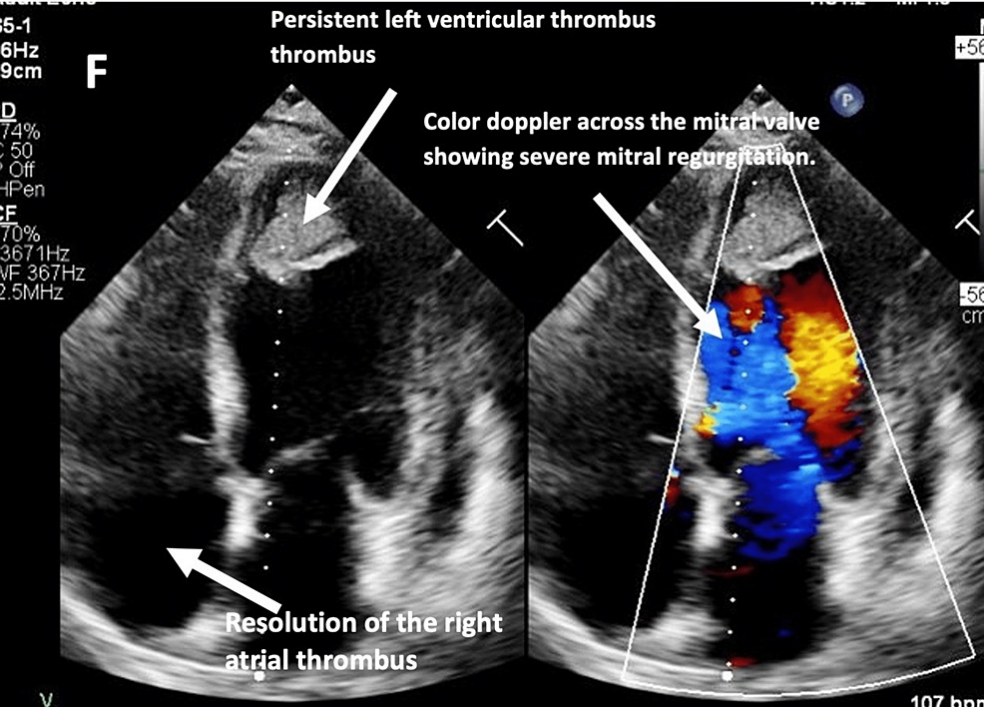
Transthoracic echocardiogram apical four-chamber view with color Doppler in the right picture showing interval resolution of the RA thrombus, with no change in the LV thrombus size along with severe mitral regurgitation (arrows). LV: left ventricle, RA: right atrium

TTE was repeated and revealed interval resolution of the RA thrombus, with no change in the LV thrombus size and persistent reduced LV and RV systolic function (Figure 8 and Video 3, 4).

**Video 3:** Transthoracic echocardiogram parasternal long-axis view showing interval resolution of the RA thrombus, with no change in the LV thrombus size. LV: left ventricle, RA: right atrium

**Video 4:** Transthoracic echocardiogram apical four-chamber view showing interval resolution of the RA thrombus, with no change in the LV thrombus size. LV: left ventricle, RA: right atrium

**Figure 9:**
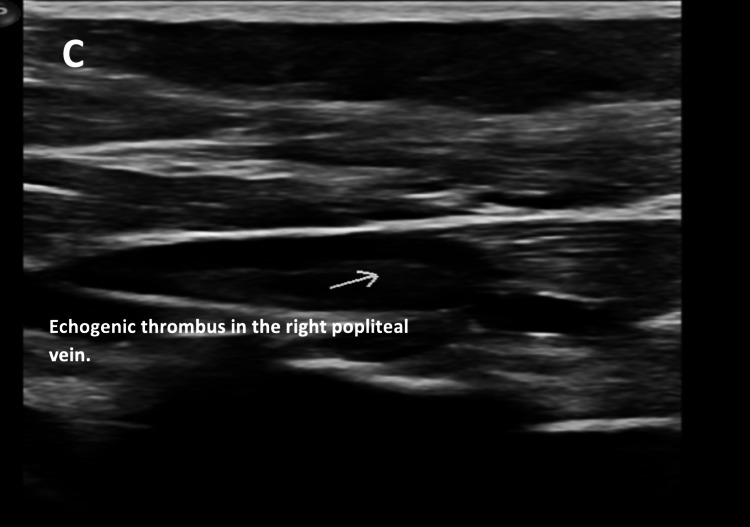
Subacute right popliteal subacute DVT (arrow). DVT: deep venous thrombosis

A lower limb venous duplex was done, which revealed acute occlusive DVT in the right peroneal vein and subacute non-occlusive DVT in the right popliteal vein (Figure 9). In these settings, antibiotics were discontinued, and therapeutic anticoagulation was continued.

In the subsequent days, heparin infusion was stopped, and apixaban was started. Heart failure treatment was optimized; losartan, empagliflozin, and metoprolol succinate were added gradually. A cardiac magnetic resonance imaging (MRI) revealed findings suggestive of non-ischemic cardiomyopathy. Left heart catheterization was done to rule out ischemic etiology and revealed angiographically normal coronary arteries.

The patient was then discharged after improvement of the symptoms with scheduled follow-up appointments with cardiology and hematology clinics.

## Discussion

This rare case demonstrates an unusual association between DIC and non-ischemic cardiomyopathy, biventricular thrombi, extensive DVT, and PE. In comparison, there are multiple prior reports for DIC with CHF and LV thrombus [4,5]. However, our case differs from previous reports regarding the presence of biventricular thrombi and extensive DVT/PE. Thus, this is the first reported case in which biventricular thrombi in non-ischemic cardiomyopathy induced DIC.

Usually, the leading presentation of DIC is acute bleeding. However, it also can be presented in the setting of newly diagnosed heart failure with consumption coagulopathy and LV thrombus [6]. Identifying the initial triggers of DIC is critical for successful DIC management. DIC development in heart failure patients results from multifactorial processes. Cugno et al. [7] suggested that the activation of the neuroendocrine system in CHF is the main triggering factor for coagulation cascade activation, endothelial dysfunction, and increased inflammatory biomarkers. Belov et al. [4] proposed that liver dysfunction has a significant role in developing DIC. Because the liver is the powerhouse for procoagulants and anticoagulants, in this patient, profound liver congestion secondary to biventricular failure and severe tricuspid regurgitation contributed to synthetic liver dysfunction and predisposed to DIC development [8]. Ventricular thrombus formation can result from stasis from heart failure, which in turn consumes coagulation factors locally, contributing to overt DIC. Initially, the hematologic derangements prohibited anticoagulation; therefore, thrombectomy to remove the right-sided thrombi and PE was a priority for this patient. Thrombolytic agents, surgical thrombectomy, and anticoagulation are also reasonable treatment options.

Optimizing hemodynamic status is fundamental in DIC management in acute heart failure. Our patient was presented with volume overload but not poor perfusion, so diuretics were essential to restore volume status and decrease liver congestion. Anticoagulation was initially held until the correction of DIC. The patient received 10 units of cryoprecipitate for persistent low fibrinogen levels. She also received an inferior vena cava filter to prevent further showers of PE. A few days later, DIC was corrected; she underwent a left heart catheterization revealing normal coronaries and then was discharged on apixaban and heart failure medication.

## Conclusions

Patients with severe cardiomyopathy can develop multiple thrombotic events with a subsequent DIC picture. Our patient presented with heart failure symptoms and was found to have biventricular thrombi, bilateral PE, and extensive DVT. Early diagnosis of DIC and cardiac thrombi is essential to the successful implementation of proper care, which includes thrombectomy, optimization of heart failure medications, and anticoagulation. This will lead to improved patient outcomes. Our patient underwent a thrombectomy for the RA thrombus and the PE, along with the placement of an IVC filter. She was placed on therapeutic oral anticoagulation and guideline-directed medical therapy (GDMT) for heart failure.
